# A subset of mobilized human hematopoietic stem cells express germ layer lineage genes which can be modulated by culture conditions

**DOI:** 10.1186/s13287-018-0858-5

**Published:** 2018-05-02

**Authors:** Tapas Makar, Vamshi K. Nimmagadda, Poornachander R. Guda, Brian Hampton, Weiliang Huang, Maureen A. Kane, Paul S. Fishman, Bernard Pessac, Christopher T. Bever, David Trisler

**Affiliations:** 1 Department of Neurology, University of Maryland School of Medicine, Baltimore, MD 21201 USA; 2 Multiple Sclerosis Center of Excellence, East VA Maryland Health Care System, Baltimore, MD 21201 USA; 3 VA Maryland Health Care System, Baltimore, 21201 USA; 4 Protein Analysis Laboratory, Center for Vascular and Inflammatory Diseases, University of Maryland School of Medicine, Baltimore, MD 21201 USA; 5 University of Maryland School of Pharmacy, Department of Pharmaceutical Sciences, Baltimore, MD 21201 USA; 6 Mass Spectrometry Center, University of Maryland School of Pharmacy, Baltimore, MD 21201 USA; 7 CNRS UMR 8118, Université Paris Descartes, 45 rue des Saints-Pères, 75006 Paris, France; 8 Académie Nationale de Médecine, 16 rue Bonaparte, 75006 Paris, France

**Keywords:** CD34^+^ stem cells, Human bone marrow stem cell, ESC genes, Lineage genes, Pancreas, Insulin

## Abstract

**Background:**

Adult bone marrow contains stem cells that replenish the myeloid and lymphoid lineages. A subset of human and mouse CD34^+^ bone marrow stem cells can be propagated in culture to autonomously express embryonic stem cell genes and embryonic germ layer lineage genes. The current study was undertaken to determine whether these CD34^+^ stem cells could be obtained from human blood, whether gene expression could be modulated by culture conditions and whether the cells produce insulin.

**Methods:**

Human peripheral blood buffy coat cells and mobilized CD34^+^ cells from human blood and from blood from C57Bl/6 J mice were cultured in hybridoma medium or neural stem cell induction medium supplemented with interleukin (IL)-3, IL-6, and stem cell factor (SCF). Changes in mRNA and protein expression were assessed by Western blot analysis and by immunohistochemistry. Mass spectrometry was used to assess insulin production.

**Results:**

We were able to culture CD34^+^ cells expressing embryonic stem cell and embryonic germ layer lineage genes from adult human peripheral blood after standard mobilization procedures and from mouse peripheral blood. Gene expression could be modulated by culture conditions, and the cells produced insulin in culture.

**Conclusion:**

These results suggest a practical method for obtaining large numbers of CD34^+^ cells from humans to allow studies on their potential to differentiate into other cell types.

**Electronic supplementary material:**

The online version of this article (10.1186/s13287-018-0858-5) contains supplementary material, which is available to authorized users.

## Background

Adult bone marrow contains stem cells that replenish the myeloid and lymphoid lineages throughout life [1, 2]. In addition to the expected hematopoietic stem cell genes CD34, cKit, and CD45 [3], a subset of these adult human and mouse CD34^+^ bone marrow stem cells express the embryonic stem cell genes Oct-4, Sox-2, LIN-28 [4], and embryonic germ layer lineage genes such as ectodermal neuronal (neurofilament, tubulin) and oligodendroglial (NG-2, MOG, PLP, MBP (human)), mesodermal cardiac (MEF, troponin C, MLC-2), and endodermal pancreatic (Pdx-1, Ptf1α, Ngn-3, FoxA2, Sox17, CXCR4, Hnf1B, Hnf4A, Hnf6, Nkx2.2, Pax-6, GLUT2, glucokinase, proinsulin and insulin (human)) and intestinal (villin) genes [5]. A cell culture method was developed to propagate a pure population of this subset of CD34^+^ stem cells [6]. The gene expression profile suggested that this subset of CD34^+^ stem cells might have characteristics of embryonic stem cells and might be capable of developing into cell types other than myeloid and lymphoid cells. To test this possibility, adult mouse CD34^+^ bone marrow stem cells were expanded in culture, labeled, and inserted into mouse blastocysts to determine their fates in the mice that developed. It was found that the labeled cells formed multiple organs in chimeric mice suggesting that, when placed in the proper conditions, the cells could differentiate into multiple different lineages [7]. While our early animal studies [6, 8] and those of others and a series of case reports suggested that adult CD34^+^ stem cells could transdifferentiate to nonhematogenous cell types when transplanted into adults, this remains controversial because of findings of fusion of CD34^+^ stem cells with tissue-specific cells (reviewed in Porada et al. [9]) and findings suggesting that CD34 is a common marker of diverse progenitors (reviewed in Sidney et al. [10]). The current study was undertaken to determine whether CD34^+^ stem cells that could be induced to express embryonic germ layer lineage genes could be obtained from mobilized human peripheral blood, whether the cells so generated expressed proteins not expected in hematogenous stem cells in conditions where cell fusion was not possible, and whether gene expression could be modulated by culture conditions.

## Methods

### Cells

Human buffy coat cells and CD34^+^ cells from peripheral blood were purchased from ALLCELLS (Alameda, CA, USA). Human buffy coat cells were obtained by differential centrifugation of blood from normal donors. The human CD34^+^ cells had been mobilized by the company by injecting donors with five daily injections of granulocyte macrophage colony-stimulating factor (GCSF). Blood was then collected and CD34^+^ cells were obtained by flow cytometry. We received 1 × 10^6^ CD34^+^ hematopoietic stem cells pooled from multiple donors that were put directly into culture as described below.

Human bone marrow cells were purchased from a commercial vendor (Lonza, Inc., Walkersville, MD, USA) and separated by differential centrifugation as described previously [6]. CD34^+^ cells were grown as described below.

Blood was obtained from C57Bl/6 J mice by cardiac puncture, buffy coats obtained by differential centrifugation, and CD34^+^ stem cells grown as described below. All procedures were performed in accordance with the approved University of Maryland School of Medicine Institutional Animal Care and Use Committee Protocol. National Institutes of Health guidelines for animal treatment and care were followed assiduously.

### Cell culture

Human CD34^+^ stem cells were cultured from bone marrow-derived buffy coat or in situ mobilized CD34^+^ bone marrow stem cells by culture in GIBCO Hybridoma serum-free medium and GIBCO protein-free medium each supplemented with 10 ng/ml human interleukin (IL)-3, 5 ng/ml human IL-6, 10 ng/ml human stem cell factor (SCF), and β-mercaptoethanol in T75 tissue culture flasks as reported previously [6]. Only suspended cells were retained at each passage; cells adherent to the flask were discarded. At each passage 2 × 10^6^ suspension cells were replated in a new T75 flask. No feeder cells or matrix were employed.

To investigate whether culture conditions could be used to modulate gene expression in mobilized human CD34^+^ stem cells obtained from blood, after 14 days of culture in serum-free hybridoma medium supplemented with IL-3, IL-6, SCF, and β-mercaptoethanol the human peripheral blood CD34^+^ cells were switched to STEMdiff Neural Induction medium (STEMCELL Technologies, Vancouver, BC, Canada), a medium developed to induce human embryonic stem cells and human induced pluripotent stem cells to become neural stem cells. Cells were cultured in T75 flasks without feeder cells or matrix and remained viable until used on day 4 of culture.

To investigate whether the insulin found in the culture medium of CD34^+^ stems cells was produced by the stem cells (and not carried over from the medium), cells were cultured using the SILAC metabolic labeling system (ThermoFisher Scientific) containing ^13^C-leucine. Culture supernatants were collected and centrifuged for analysis by mass spectrometry.

Mouse CD34^+^ stem cells were cultured from peripheral blood buffy coats in 10 ml GIBCO DMEM/10% fetal bovine serum supplemented with 10 ng/ml mouse IL-3, 5 ng/ml mouse IL-6, 10 ng/ml mouse SCF, and β-mercaptoethanol, the growth medium developed to grow CD34^+^ hematopoietic progenitors from adult mouse bone marrow. The cells were cultured in T75 tissue culture flasks as reported [6] with only suspension cells being retained at each passage with 2 × 10^6^ suspension cells being replated in a new T75 flask. No feeder cells or matrix were employed.

### Quantitative polymerase chain reaction

Quantitative polymerase chain reaction (PCR) was carried out using total RNA according to the methods given in the Additional file 1 with the primers listed in Table 1.

**Table 1:** Primers used for quantitative polymerase chain reaction

	Forward	Reverse
CD34 (99 bp)	5’GCAGGTAAACTCCTGTCCTTTA3’	5’TTCTCCAGACCTTGGCTTTC3’
Neurofilament-H (149 bp)	5’GAGGGTCTCCTCTGACG3’	5’CTTGGCAGTGAGAGGGT3’
Oct 4 (96 bp)	5’CCCTCTAAGGAGTATCCCTGAA3’	5’CTCAAAGCATCTTCTCCCTCTC3’
Pdx 1 (109 bp)	5’TCCTCTTCCTCCTCCTCTTTC3’	5’GTAGTGAAGTGTGCAGCTAGAG3’
Pax 6 (98 bp)	5’GCGGAAGCTGCAAAGAAATAG3’	5’GGGCAAACACATCTGGATAATG3’
MOG (107 bp)	5’TCTCTAGGGTGGTTCATCTCTAC3’	5’TCCCTCACCAATAGCATCTTTC3’
Tuj-1 (112 bp)	5’CGAAGCCAGCAGTGTCTAAA3’	5’GGAGGACGAGGCCATAAATAC3’
cKit (109 bp)	5’GATTCCCAGAGCCCACAATAG3’	5’GGTGGCCCAGATGAGTTTAG3’
Villin (95 bp)	5’GCTGTCTGCCCTAGTTCATATC3’	5’TGGGCATGGGTGCTTTATT3’
SOX-2 (76 BP)	5’AGACGCTCATGAAGAAGGATAAG3’	5’CCGCTCGCCATGCTATT3’
LIN-28 (97 BP)	5’CAGAGTGGAGAAAGTGGGAATAG3’	5’CTAGAGGGAAGAAAGGGTGATG3’
NG2 (102 bp)	5’AACCAGGGTAACCTCCTACA3’	5’TCCTTCTCCTTGCCCTCTTA3’
PLP (111 bp)	5’CTCCAACCTTCTGTCCATCTG3’	5’ATGAAGGTGAGCAGGGAAAC3’
MBP	5’GAAGGCCAGAGACCAGGATT3’	5’AATTTGGAAAGCGTGCCCT3’
GCK	5’CCAACGGGGCCATGAATATG3’	5’TCCTTGCTTTGTCCCTCCAT3’
PTF 1a (75 bp)	5’AGCAGGACACTCTCTCTCAT3’	5’CAGACTTTGGCTGTTCGGATA3’
GLUT2	5’TGGCCATTACTAACACGCATTG3’	5’TGCTAAGCTTTTGGGACCCA3’
Proinsulin	5’AGATCACTGTCCTTCTGCCA3’	5’CGCACAGGTGTTGGTTCA3’
Insulin	5’TCAGAAGAGGCCATCAAGCA3’	5’TGGCAGAAGGACAGTGATCT3’
GAPDH (96 bp)	5’TCTTTCTTTGCAGCAATGCC3’	5’CCATGAGTCCTTCCACGATAC3’
β-Actin (92 bp)	5’CTTCCTTCCTGGGCATGG3’	5’GTACAGGTCTTTGCGGATGT3’

### Immunocytochemistry

Immunocytochemistry was carried out by standard methodology using the following antibodies: CD34 (Pharmingen, 553731), cKit (Cymbus Biotechnology, CBL1359), CD45 (Pharmingen, 553076), Oct-4 (Santa Cruz, sc-9081), Sox-2 (Santa Cruz, sc-20088), LIN28 (Santa Cruz, sc-67266), Pax-6 (Santa Cruz, sc-11357), neurofilament H (Sternberger Monoclonals, SMI 312), Tuj1 (Covance, PRB435P and MMS435P), PLP (Chemicon, MAB388), NG2 (Chemicon, AB5320), MBP (from BP), MOG (Millipore, MAB345), Pdx1 (Chemicon, AB3243), Ptf1α (R&D Systems, AF6119), glucokinase (Santa Cruz, sc7908), insulin A (Santa Cruz, sc-7839), insulin B (Santa Cruz, sc-7838), and villin (Santa Cruz, sc-7672).

### Immunofluorescence staining

Immunofluorescence staining was performed according to the methods given in the Supplemental Materials and Methods (Additional file 1).

### Western blot analysis

Western blot analyses were conducted via standard methodology using the antibodies listed above in section 2.4 (Immunocytochemistry).

### Mass spectrometry

#### Analysis of insulin peptides from human mobilized CD34^+^ stem cells grown in protein-free medium

Mass spectrometry was performed by the Protein Analysis Laboratory, Center for Vascular and Inflammatory Diseases, University of Maryland School of Medicine. The proteins from the conditioned media were captured by solid-phase extraction using TARGA C18 MicroSpin tips (The Nest Group, Southborough, MA, USA). After acidifying the conditioned media with trifluoroacetic acid to a final concentration of 0.1%, it was passed through the MiniSpin tips, washed with 10 column volumes of 0.1% trifluoroacetic acid, and proteins were eluted with 70% acetonitrile containing 0.1% trifluoroacetic acid. The eluted fraction was evaporated to dryness in a centrifugal evaporator and subsequently dissolved in 100 μl 100 mM triethylammonium bicarbonate, pH 8.5, containing 2% sodium deoxycholate. Disulfide bonds were reduced with 5 mM dithiothreitol at 37 °C for 30 min followed by alkylation of the sulfhydryl groups with chloroacetamide at room temperature in the dark. After neutralization of residual chloroacetamide by the addition of DTT, proteins were digested overnight at 37 °C with Trypsin/LysC (Promega, Madison, WI, USA) at a final concentration of 12.5 ng/μl of the enzyme mixture. The digested sample was acidified with trifluoroacetic acid to a final concentration of 1% and the precipitated sodium deoxycholate was removed by centrifugation at 10,000 × g for 5 min. The peptides were recovered from the supernatant by solid-phase extraction using the TARGA C18 MicroSpin tips as described above and subsequently analyzed by liquid chromatography tandem mass spectrometry (MS/MS). Peptides were separated by reversed phase high-performance liquid chromatography (HPLC) on an in-house pulled-tip fused silica (Molex PolyMicro Technologies) capillary column (0.1 mm × 150 mm) packed with TARGA C18 3 μ stationary phase connected to a Surveyor HPLC pump (Thermo Scientific) operated at 200 μl/min and set up to deliver a split flow to the capillary column at ~ 0.45 μl/min. Electrospray was initiated by application of 1800 V at the head of the column via a liquid junction. Gas phase ions were analyzed in an LTQ Orbitrap mass spectrometer (Thermo Scientific) using a duty cycle of one full scan followed by MS/MS scans on up to 12 of the most abundant ions, above a preset threshold, observed in the preceding full scan. These data were analyzed with Proteome Discoverer 1.4 (Thermo Scientific) using the Sequest HT search engine to match acquired spectra against a human subset of the Uniprot protein sequence database, which included sequences of common protein contaminants. Percolator was used to validate peptide-spectrum matches at a *Q* value [11] (false discovery rate (FDR)) of 0.01.

#### Analysis of insulin peptides labeled with ^13^C-leucine from human mobilized CD34^+^ stem cells grown in SILAC medium

Mass spectrometry was performed at the University of Maryland School of Pharmacy Mass Spectrometry Center. Tryptic peptides were separated on a Waters nanoACQUITY UPLC system with a 20-cm ACQUITY UPLC M-Class CSH C18 column by a 3–43% acetonitrile gradient in 0.1% formic acid over 180 min at a flow rate of 400 nL/min, and were analyzed on a coupled Thermo Scientific Orbitrap Fusion Tribrid mass spectrometer as described [12]. Tandem mass spectra were searched against human insulin chain A and chain B sequences using SEQUEST HT algorithm with a precursor tolerance of 5 ppm and a product tolerance of 0.5 Da. ^13^C-labeled leucine was treated as a variable modification, and cysteine carbamidomethylation was treated as a fixed modification.

## Results

### A subset of mobilized human and mouse CD34^+^ stem cells grow exponentially in vitro

**Fig. 1:**
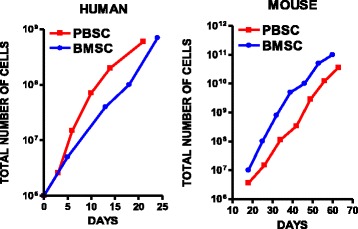
Human and mouse mobilized CD34^+^ bone marrow stem cells grow exponentially in vitro. Mobilized human CD34^+^ peripheral blood stem cells (PBSC) grew exponentially in vitro at the same rate as human CD34^+^ cells in bone marrow (BMSC). Similarly, mouse CD34^+^ cells from peripheral blood (PBSC) grew exponentially in vitro at the same rate as human CD34^+^ cells in bone marrow (BMSC). The results are shown for human and mouse cells from one of three experiments, each of which gave similar results

We determined the growth rates of mobilized human peripheral blood CD34^+^ stem cells and in situ bone marrow CD34^+^ stem cells. The mobilized CD34^+^ stem cells from peripheral blood grew exponentially at the same rate as CD34^+^ cells from adult human bone marrow (Fig. 1). The slopes of the growth curves for both human bone marrow CD34^+^ cells and human mobilized peripheral blood CD34^+^ cells were equivalent. Similarly, in the adult mouse, the CD34^+^ stem cells in C57Bl/6 J adult mouse peripheral blood grew exponentially at the same rate as CD34^+^ cells from adult C57Bl/6 J bone marrow (Fig. 1). The slopes of the growth curves for both mouse bone marrow CD34^+^ cells and mouse peripheral blood CD34^+^ cells were indistinguishable.

### Differences in CD34^+^ stem cells between human and mouse peripheral blood

We were able to culture CD34^+^ stem cells from mouse peripheral blood buffy coat, but we were not able to grow CD34^+^ bone marrow stem cells from commercial human nonmobilized blood buffy coat or from purified human nonmobilized peripheral blood mononuclear cells. We were able to culture CD34^+^ stem cells from mobilized human peripheral blood (Fig. 1). The CD34^+^ stem cell cultures from mobilized human peripheral blood differed from those obtained from the mouse in that, while the latter contained a single spherical cell morphology, the former contained four morphological phenotypes: one cell type that was adherent to the plastic flask, and three cell types that grew in suspension—a spherical cell, a cone-shaped cell, and a minute cell. All four cell types persisted throughout the culture period, although only the nonadherent cells were passaged in culture. The three nonadherent subtypes were harvested for analysis in the experiments that followed.

### Gene expression by a subset of mobilized human peripheral blood CD34^+^ cells

**Fig. 2:**
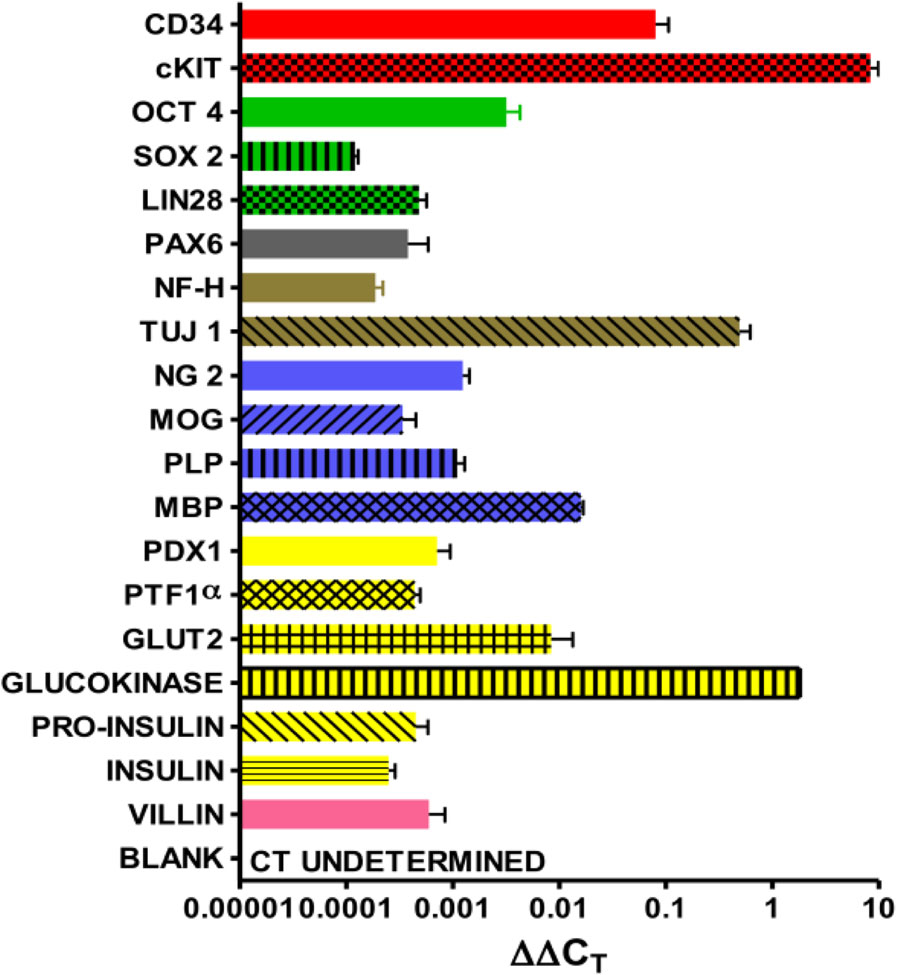
Human mobilized CD34^+^ bone marrow stem cells express mRNAs of embryonic stem cell genes and embryonic germ layer lineage genes. Quantitative PCR analysis of mRNA expression by the human mobilized CD34^+^ stem cells revealed expression of hematopoietic stem cell genes (red bars), ESC genes (green bars), neural stem cell genes (gray bar), neuronal genes (brown bars), oligodendrocyte genes (blue bars), pancreatic genes (yellow bars), and intestinal genes (pink bar). The results shown are from one of two experiments with each sample run in triplicate

Gene expression by the mobilized CD34^+^ cells from human peripheral blood, grown in culture for 15 days, was examined by quantitative PCR. The cultured human CD34^+^ cells expressed: the hematopoietic stem cell genes CD34 and cKit; the embryonic stem cell genes Oct-4, Sox-2, and Lin28; the neural stem cell gene Pax-6; the neuronal genes neurofilament H and tubulin (Tuj-1); the oligodendroglial genes NG2, MOG, PLP, and MBP; the pancreatic genes Pdx1, Ptf1a, GLUT2, glucokinase, proinsulin, and insulin; and the intestinal gene villin at the mRNA level (Fig. 2).

### Western blot analysis of protein expression by a subset of human mobilized CD34^+^ bone marrow stem cells

**Fig. 3:**
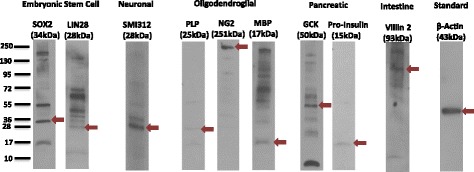
Human mobilized CD34^+^ bone marrow stem cells express proteins of embryonic stem cell genes and embryonic germ layer lineage genes. Western blot analyses revealed that the human mobilized CD34^+^ stem cells express embryonic stem cell, neuronal, oligodendroglial, pancreatic, and intestinal proteins. Each sample was run twice and the results are representative

After 21 days of culture, the mobilized human peripheral blood CD34^+^ cells were examined by Western blot analysis (Fig. 3). The mobilized cells expressed embryonic stem cell (ESC) Sox2 and LIN28 proteins, the neuronal neurofilament protein SMI312, oligodendroglial PLP, NG2, and MBP proteins, pancreatic glucokinase and preproinsulin protein, and intestinal villin protein (Fig. 3). A difference between human and mouse mobilized CD34^+^ stem cells is that human CD34^+^ cells express MBP and insulin mRNA and protein, whereas mouse CD34^+^ cells do not.

### Human and mouse mobilized CD34^+^ stem cells uniformly express ESC and germ layer lineage genes in vitro

**Fig. 4:**
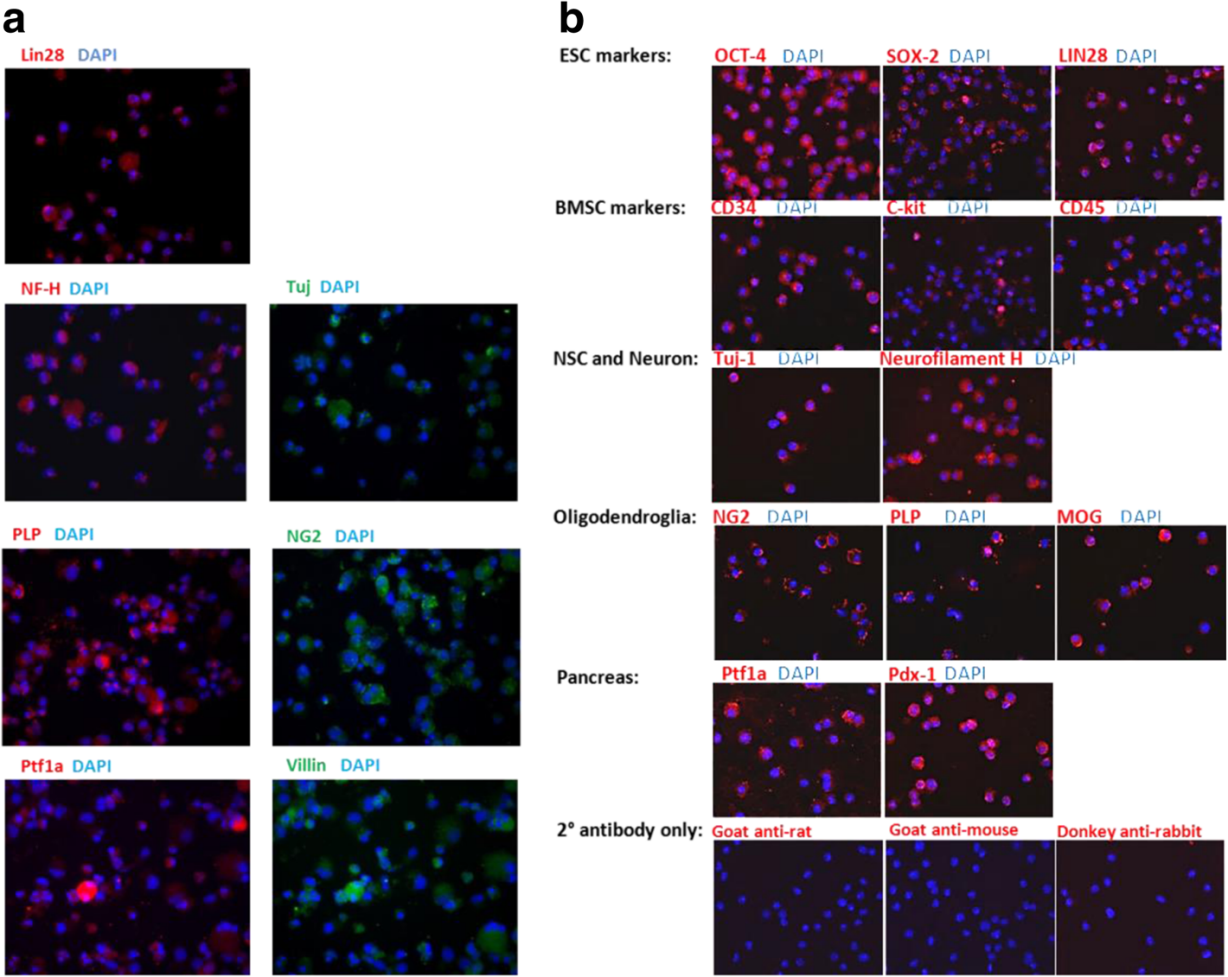
All human and mouse mobilized CD34^+^ bone marrow stem cells exhibit embryonic stem cell (ESC) and germ layer lineage proteins in vitro. Immunocytochemistry of mobilized human (**a**) and mouse (**b**) CD34^+^ cells in culture show that all human and mouse cells expressed ESC and germ layer lineage proteins. DAPI (blue) cell nuclear staining indicates all of the nucleated cells in each field. The results given for human are representative of two experiments, and for mouse of three experiments. BMSC, bone marrow stem cell; NSC, neural stem cell

Immunocytochemistry revealed that all human CD34^+^ cells in vitro expressed the embryonic stem cell protein LIN28. Double labeling showed that the CD34^+^ cells also exhibited neuronal neurofilament H and tubulin (Tuj1). Double labeling also demonstrated that the CD34^+^ cells expressed oligodendrocyte NG2 and PLP. In addition, double labeling revealed that the human CD34^+^ cells express pancreatic Ptf-1a and intestinal villin. In all cases, the four human CD34^+^ cell morphological phenotypes (adherent, suspension spherical, conical, and minute) exhibited staining for each protein (Fig. 4a). The phenotype of mouse CD34^+^ cells grown from blood was the same as that for those CD34^+^ cells grown from bone marrow for all representative genes tested. Previously, we reported that mouse bone marrow cells expressed: the ESC markers Oct-4, Sox-2, and LIN28; the bone marrow stem cell markers CD34^+^, cKit, and CD45; neural stem cell (NSC) and neuronal markers Pax-6, neurofilament H, and tubulin; the oligodendroglial markers NG2, PLP, and MOG; and the pancreatic markers Pax-6, Ptf1a, and Pdx-1. These markers were reported to be present in CD34^+^ cells cultured from mouse bone marrow [5, 6]. Here, we show that these genes are also expressed in CD34^+^ cells cultured from mouse peripheral blood (Fig. 4b). Thus, in mobilized human CD34^+^ stem cells and mouse peripheral blood CD34^+^ stem cells cultured in vitro, immunocytochemistry showed that all cells in culture expressed each of the proteins tested.

### Gene expression in a subset of human peripheral blood CD34^+^ can be modulated by culture conditions

**Fig. 5:**
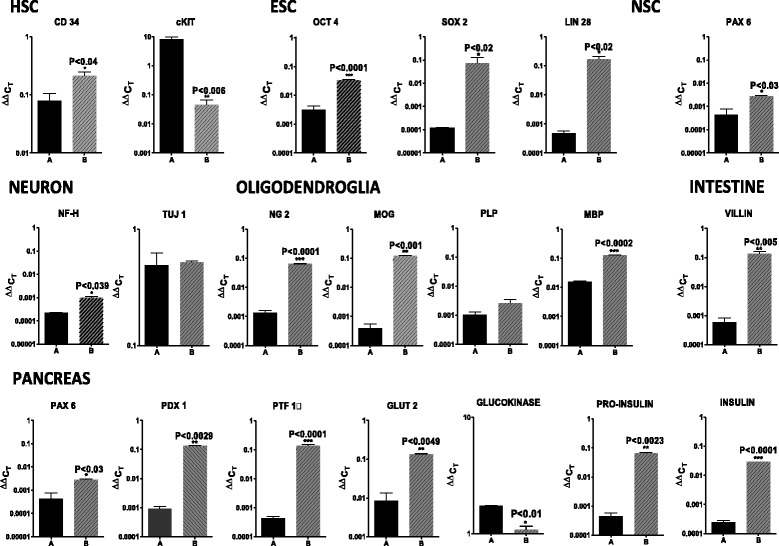
Human mobilized CD34^+^ bone marrow stem cell expression of embryonic stem cell and embryonic germ layer lineage mRNAs is regulated by in vitro culture conditions. Quantitative PCR analyses of gene expression (mRNA levels) by human mobilized CD34^+^ stem cells revealed mRNA regulation by culture conditions. Black bars are values for cells grown in our CD34^+^ bone marrow stem cell growth medium and gray bars are values for STEMdiff medium. Student’s *t* test *P* values are shown for each mRNA. The values given are the averages of triplicate samples. ESC, embryonic stem cell; HSC, hematopoietic stem cell; NSC, neural stem cell

After 14 days in culture in serum-free hybridoma medium supplemented with IL-3, IL-6, SCF, and β-mercaptoethanol, the human peripheral blood CD34^+^ cells were switched to STEMdiff Neural Induction medium (STEMCELL Technologies). STEMdiff medium was developed to induce human embryonic stem cells and human induced pluripotent stem cells to become neural stem cells. We compared mRNA expression of human mobilized peripheral blood CD34^+^ cells after 4 days of culture in STEMdiff Neural Induction medium by quantitative PCR (Fig. 5). Figure compares gene expression in serum-free hybridoma medium supplemented with IL-3, IL- 6, SCF, and β-mercaptoethanol with gene expression in STEMdiff Neural Induction medium for 4 days. The level of hematopoietic stem cell (HSC) CD34 expression was virtually unchanged in STEMdiff medium, whereas cKit was markedly reduced 5000-fold. The ESC marker Oct-4 was elevated 20-fold in STEMdiff medium, Sox-2 was increased 1000-fold, and LIN28 was increased 500-fold. The NSC marker Pax-6 was increased 10-fold in STEMdiff medium. Neuronal neurofilament H was increased 50-fold, whereas tubulin was virtually unchanged in STEMdiff medium. Oligodendroglial NG-2 was increased 350-fold, MOG was increased 500-fold, and PLP was virtually unchanged, while MBP increased 10-fold in STEMdiff medium. Intestinal villin was increased 500-fold in STEMdiff medium. Pancreatic Pax6 was increased 10-fold, Pdx-1 was increased 200-fold, and Ptf1a was increased 500-fold, while GLUT-2 increased 10-fold, glucokinase was virtually unchanged, proinsulin increased 100-fold, and insulin increased 100-fold in STEMdiff medium. In summary, culture of human CD34^+^ stem cells in the neural induction medium led to increases in the expression of the genes studied except for cKIT and glucokinase, which were decreased, and Tuj1 and PLP which were not significantly different.

### A subset of human mobilized CD34^+^ stem cells synthesize insulin

**Fig. 6:**
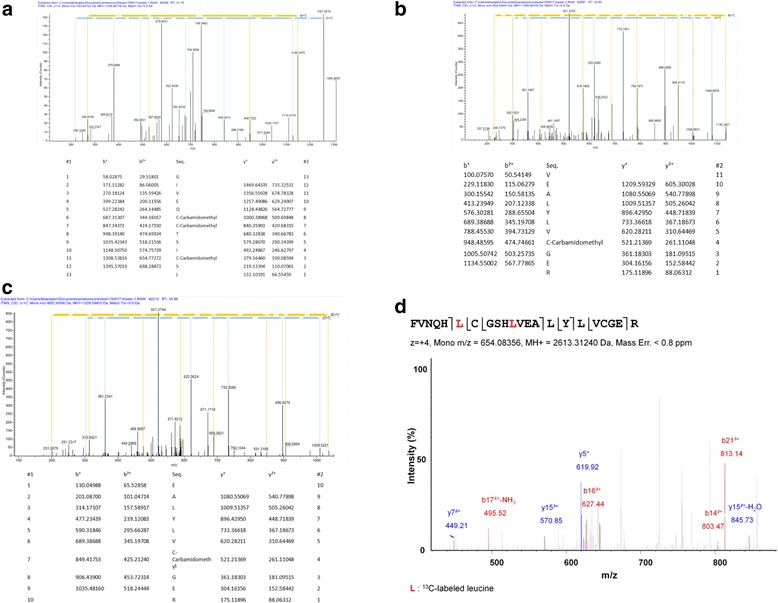
Mobilized human CD34^+^ bone marrow stem cells synthesize and release insulin in culture. Culture supernatants were analyzed by mass spectrometry. **a** Tracing of insulin alpha chain peptide GIVEIQCCTSICSL present in human mobilized CD34^+^ stem cell conditioned protein-free culture medium. **b** Tracing of insulin beta peptide VEALYLVCGER present in human mobilized CD34^+^ stem cell conditioned protein-free culture medium. **c** Tracing of insulin beta chain peptide EALYLVCGER present in human mobilized CD34^+^ stem cell conditioned protein-free culture medium. **d** Tracing of insulin beta peptide FVNQHLCGSHLVEALYLVCGER labeled with ^13^C-leucine present in human mobilized CD34^+^ stem cells cultured in ^13^C-leucine containing SILAC metabolic labeling medium. Each result shown is from a single experiment

Mobilized human CD34^+^ stem cells that were growing in serum-free GIBCO Hybridoma serum-free cell culture medium, supplemented with human IL-3, IL- 6, SCF, and β-mercaptoethanol, were washed twice in 15 ml protein-free medium to remove the insulin present in the serum-free medium. The cells were then cultured in GIBCO protein-free medium supplemented with IL3, IL 6, SCF, and β-mercaptoethanol. After 5 days in culture the cells were removed from the medium by centrifugation. The cell culture medium was then analyzed by mass spectrometry for insulin. Three peptides of human insulin were revealed by mass spectrometry: two beta chain peptides, VEALYLVCGER (Fig. 6a) and EALYVCGER (Fig. 6b), and one alpha-chain peptide, GIVEQCCTICSL (Fig. 6c). To determine whether the insulin that was detected was synthesized by the cells and not carried over from the medium, medium from cells cultured in metabolic labeling medium with ^13^C-leucine was analyzed by mass spectrometry. The beta chain peptide FVNQHLCGSHLVEALYLVCGER was identified with the label at the first and second leucines. These results suggest the CD34^+^ cells synthesize insulin under the conditions described.

## Discussion

The potential of stem cells as cell replacement therapy for degenerative disease and other types of tissue injury has been complicated by the ethical issues surrounding the use of embryonic stem cells [13], the histocompatibility issues of tissue-specific stem cells from other individuals [14], the difficulty obtaining large quantities of inducible pluripotent stem cells [15], and teratoma formation by both embryonic and inducible pluripotent stem cells [16]. Early reports from mouse studies and studies of human transplant recipients suggesting the possibility that hematopoietic stem cells could transdifferentiate into cells of other types including neural cells [17–21], cardiac cells [22–24], hepatic cells [25–28], and pancreatic cells [29, 30] sparked interest in hematogenous stem cells for cell replacement therapy. Subsequently a population of very small embryonic-like (VSEL) stem cells was identified as a possible circulating pluripotent stem cell [31, 32]. More recently, this enthusiasm has been tempered by evidence that at least some of the observations were due to cell fusion [9], the presence of CD34 antigen on progenitor cells not of hematogenous origin [10], and the failure to confirm the findings regarding VSEL stem cells [33]. While we were able to exclude cell fusion as an explanation in our earlier work suggesting that CD34^+^ stem cells could transdifferentiate into multiple lineages when placed into mouse blastocysts [7], in studies showing transdifferentiation into oligodendroglial cells which ensheath axons in *Shiverer* mice [8] we did not fully exclude the possibility of cell fusion. We did identify a small cell morphology in cultured cells from mobilized human blood that might correspond with the previously reported VSEL stem cells [31], but we did not attempt to separate or test the cells separately with that morphology. Therefore, it was important to demonstrate that gene expression by the CD34^+^ cells can be modulated (in this case by culture conditions) and that the CD34^+^ cells express genes characteristic of differentiated tissues (in this case insulin). The current results show both that gene expression by the CD34^+^ cells can be modulated by culture conditions and that the CD34^+^ cells can translate and transcribe the gene for insulin in conditions where cell fusion with pancreatic cells was not possible. These findings support the idea that this subpopulation of CD34^+^ stem cells might either be at a stage prior to commitment to the hematogenous lineage or be capable of transdifferentiation from that lineage to other lineages. Further work is needed to determine this.

If these CD34^+^ stem cells are shown to be capable of differentiating into other cells types, then the practical issue of their availability would be important. We have shown previously that, although the cells could be obtained from mouse bone marrow and peripheral blood, we could obtain them from human bone marrow but not peripheral blood. Our results in the current study showing that the cells can be obtained from mobilized peripheral blood would allow for a practical method for obtaining large numbers of cells should they be found useful in cell replacement therapy or for other purposes.

## Conclusions

Mobilized human CD34^+^ bone marrow stem cells constitutively express an array of ESC and embryonic germ layer lineage genes. Gene expression can be modulated by modifying culture conditions, and the cells synthesize insulin. These results suggest a practical source of autologous stem cells for studies to determine whether they might be useful in cell replacement therapy.

## Additional file

Additional file 1: Supplemental materials and methods. (DOCX 17 kb)
