# Supplementary material for: A subset of mobilized human hematopoietic stem cells express germ layer lineage genes which can be modulated by culture conditions
Source: Stem Cell Res Ther. 2018 May 2;9:127. doi: 10.1186/s13287-018-0858-5 (PMC5930965; doi:10.1186/s13287-018-0858-5)
Supplement: Supplementary file 1 — Supplemental materials and methods. (DOCX 17 kb) [file 13287_2018_858_MOESM1_ESM.docx]

**Total RNA preparation and quantitative real-time PCR:**

Total RNA was isolated using a TRIzol solution (Life technologies, USA) following manufacturer’s recommended protocol. 1 µg mRNA was used to generate cDNA by using the reverse transcriptase kit (Promega, USA). A template of cDNA was applied in the following PCR reactions (Step-one plus, Applied Biosciences, USA). β-actin was used as housekeeping gene. The nucleotide sequences of primers and reaction conditions are listed below:

CD34 (99 bp) Forward 5’GCAGGTAAACTCCTGTCCTTTA3’ Reverse 5’TTCTCCAGACCTTGGCTTTC3’; Neurofilament-H (149 bp) Forward 5’GAGGGTCTCCTCTGACG3’ Reverse 5’CTTGGCAGTGAGAGGGT3’;

Oct 4 ( 96 bp ) Forward 5’CCCTCTAAGGAGTATCCCTGAA3’ Reverse 5’CTCAAAGCATCTTCTCCCTCTC3’;

Pdx 1 (109 bp) Forward 5’TCCTCTTCCTCCTCCTCTTTC3’ Reverse 5’GTAGTGAAGTGTGCAGCTAGAG3’;

Pax 6 (98 bp) Forward 5’GCGGAAGCTGCAAAGAAATAG3’ Reverse 5’GGGCAAACACATCTGGATAATG3’; MOG (107 bp) Forward 5’TCTCTAGGGTGGTTCATCTCTAC3’ Reverse 5’TCCCTCACCAATAGCATCTTTC3’; Tuj-1 (112 bp) Forward 5’CGAAGCCAGCAGTGTCTAAA3’ Reverse 5’GGAGGACGAGGCCATAAATAC3’;

cKit (109 bp) Forward 5’GATTCCCAGAGCCCACAATAG3’ Reverse 5’GGTGGCCCAGATGAGTTTAG3’;

villin (95 bp) Forward 5’GCTGTCTGCCCTAGTTCATATC3’ Reverse 5’TGGGCATGGGTGCTTTATT3’;

SOX-2 (76 BP) Forward 5’AGACGCTCATGAAGAAGGATAAG3’ Reverse 5’CCGCTCGCCATGCTATT3’;

LIN-28 (97 BP) Forward 5’CAGAGTGGAGAAAGTGGGAATAG3’ Reverse 5’CTAGAGGGAAGAAAGGGTGATG3’;

NG2 (102 bp) Forward 5’AACCAGGGTAACCTCCTACA3’ Reverse 5’TCCTTCTCCTTGCCCTCTTA3’;

PLP (111 bp) Forward 5’CTCCAACCTTCTGTCCATCTG3’ Reverse 5’ATGAAGGTGAGCAGGGAAAC3’;

MBP Forward 5’GAAGGCCAGAGACCAGGATT3’ Reverse 5’AATTTGGAAAGCGTGCCCT3’;

GCK Forward 5’CCAACGGGGCCATGAATATG3’ Reverse 5’TCCTTGCTTTGTCCCTCCAT3’;

PTF 1a (75 bp) Forward 5’AGCAGGACACTCTCTCTCAT3’ Reverse 5’CAGACTTTGGCTGTTCGGATA3’;

GLUT2 Forward 5’TGGCCATTACTAACACGCATTG3’ Reverse 5’TGCTAAGCTTTTGGGACCCA3’;

Proinsulin Forward 5’AGATCACTGTCCTTCTGCCA3’ Reverse 5’CGCACAGGTGTTGGTTCA3’;

Insulin Forward 5’TCAGAAGAGGCCATCAAGCA3’ Reverse 5’TGGCAGAAGGACAGTGATCT3’.

GAPDH (96 BP) Forward 5’TCTTTCTTTGCAGCAATGCC3’ Reverse 5’CCATGAGTCCTTCCACGATAC3’;

B-Actin (92 BP) Forward 5’CTTCCTTCCTGGGCATGG3’ Reverse 5’GTACAGGTCTTTGCGGATGT3’

The cDNA was analyzed by quantitative PCR with SYBR Green Realtime PCR Master Mix Plus (Toyobo) on a CFX96 Touch Real-Time PCR Detection System (Bio-Rad, Hercules, CA, USA).

**Immuno fluorescence staining**

The cultured cells were placed on 15 ml tube, fixed in 4% paraformaldehyde (PFA; Sigma) for 10 min at room temperature, washed three times with phosphate-buffered saline (PBS). T cells were collected in glass slides by cytospine and treated with a permeabilizing and blocking buffer (10% donkey serums, 0.225% Triton X-100) for 1 hour at room temperature. Then, the cells were incubated with the following primary antibodies: : CD34 (1:500, Pharmingen 553731), cKit (1:200,Cymbus Biotechnology CBL1359), CD45 (1:500,Pharmingen 553076), Oct-4 (1:500,Santa Cruz sc-9081), Sox-2 (1:500,Santa Cruz sc-20088), LIN28 ( 1:200,Santa Cruz sc-67266), Pax-6 (1:200, Santa Cruz sc-11357), Neurofilament H (1:500, Biolegend-801701), Tuj1 (1:250,Covance PRB435P and MMS 435P), PLP (1:500, Chemicon MAB388), NG2 (1:250, Chemicon- AB5320), MBP (1:500, Millipore- AB9348), MOG (1:1000, Millipore -MAB345), Pdx1 (1:300, Chemicon -AB3243), Ptf1α (1:500, R&D Systems- AF6119), glucokinase (1:200,Santa Cruz sc-7908), insulin A (1:500,Santa Cruz sc-7839), insulin B (1:500,Santa Cruz sc-7838), villin (1:200, Santa Cruz sc-7672). All antibodies were diluted in antibody dilution buffer (2% donkey serum, 0.05%Triton X-100), and the cells were incubated with the antibodies overnight at 4°C. After three washing steps, the cells were incubated for 1 hour at room temperature with corresponding secondary antibodies either FITCY donkey anti-mouse, donkey anti-rabbit, and donkey anti-goat or TRITCY donkey anti-mouse, donkey anti-rabbit, and donkey anti-goat according to species the primary antibody was raised in. The cells were then washed 3 times in PBS and mounted the cover slip after adding the mounting medium containing DAPI (Vector). All cell samples were observed using an Olympus fluorescence microscope
